# Influence of trunk strength on sprint performance in swimmers: a cross-sectional analysis of torque-velocity relationships

**DOI:** 10.3389/fphys.2025.1625283

**Published:** 2025-09-23

**Authors:** Jinjin Dai, Zicheng Dai, Yahui Ding

**Affiliations:** ^1^ Department of Sports, Zhejiang Wanli University, Ningbo, China; ^2^ Sports Coaching College, Beijing Sports University, Beijing, China; ^3^ Swimming Department, Zhejiang College of Sports, Hangzhou, China; ^4^ Faculty of Physical Education, Pingdingshan University, Pingdingshan, China

**Keywords:** trunk strength, sprint performance, swimmers, freestyle, torque-velocity

## Abstract

This cross-sectional study assessed trunk strength at 60°/s and 120°/s angular velocities in swimmers and its relationship to 100-m sprint performance. Thirty-two elite swimmers (age: 19.49 ± 1.44 years; height: 177.77 ± 6.84 cm; body mass: 71.88 ± 8.50 kg) underwent isokinetic trunk testing and timed sprints. All tests demonstrated excellent reliability (ICC >0.96). Swimmers had significantly greater peak torque in extension compared to flexion (*p* < 0.01), and higher torque in left versus right rotation, though the latter was not significant. Contrary to the hypotheses, peak torque at 120°/s did not correlate more strongly with performance than at 60°/s, and rotation torque did not surpass flexion/extension metrics. After Benjamini–Hochberg FDR correction for 24 comparisons, no significant correlations remained (q < 0.05), indicating initial associations were likely confounded by sex differences. These results suggest training should emphasize inter-segmental coordination over isolated strength gains, focusing on torque transfer from trunk to extremities. Interpretation of high-velocity torque data requires caution due to potential acceleration artifacts at early peak angles (5°–7°).

## Introduction

The primary objective of competitive swimming is to efficiently traverse a set distance in the shortest time possible. Therefore, improving muscular strength for increased propulsion, maintaining streamlined alignment, and minimizing drag through optimal body positioning are vital for enhancing athletic performance in swimmers (Fig, 2005; Kibler et al., 2006). Proper alignment of the head, shoulders, trunk, pelvis, and lower limbs forms the technical foundation of swimming, and aligning these body segments in a nearly straight line reduces hydrodynamic resistance and enhances swimming efficiency (Willardson, 2007; Jia et al., 2022). Trunk muscles play a crucial role in maintaining body posture and providing active stabilization in the unstable aquatic environment (Patil et al., 2014). Insufficient strength in trunk muscles can lead to energy wastage due to compromised stabilization (Martens et al., 2013; Khiyami et al., 2022). Furthermore, maintaining a stable body position during swimming is crucial for optimizing power output from both the upper and lower limbs (Willardson, 2007; Strzala et al., 2012). Trunk muscle training is considered beneficial as it enhances stabilization, resulting in increased force production by the limbs and efficient force transmission between the trunk and extremities (Hibbs et al., 2008; Dingley et al., 2014; Weston et al., 2015). In sprint swimmers, trunk strength training is thought to induce neuromuscular adaptations such as enhanced neural activation, improved motor unit synchronization, optimized recruitment patterns, and reduced inhibitory reflexes (Strzala et al., 2012; Dingley et al., 2014), and these adaptations directly contribute to enhancing stroke efficiency and athletic performance.

However, empirical evidence supporting trunk strength’s direct impact on swimming performance is conflicted. While core training improves swim efficiency in adolescents (Karpiński et al., 2020), elite studies show minimal transfer (Keiner et al., 2021). This may stem from methodological limitations: most assessments use isometric/endurance tests ill-suited to capture velocity-specific strength adaptations crucial for sprint swimming. Trunk strength is typically measured by the number of repetitions and the load lifted (Faries and Greenwood, 2007). The endurance of the anterior, posterior, and lateral trunk muscles is assessed using trunk flexion, trunk extension, and right and left side bridge tests (Reed et al., 2012; Shamsi et al., 2016). Most studies have evaluated the maximal isometric strength and endurance of the trunk, with little attention paid to the load, force, and power-velocity relationships (Zemkova and Zapletalova, 2022). Therefore, further research is needed to address this gap in the literature and investigate strength and power-related measures within cross-sectional and intervention studies. The lack of specificity in many dryland strength training programs is frequently cited as a reason for their limited effectiveness in improving swimming performance (Girold et al., 2007). Critically, no study has examined isokinetic trunk strength which quantifies torque-velocity profiles in relation to segmental swimming performance, despite its potential to reveal sport-specific neuromuscular adaptations.

This gap is compounded by a lack of sport-specific validation. Static endurance testing is well-suited for assessing postural stability in endurance-oriented sports, while Isokinetic assessmen may be more appropriate for disciplines emphasizing strength (Pérez-Olea et al., 2018). Isokinetic testing provides precise measurements of muscle strength under controlled conditions, including angular velocities, contraction types, and motion ranges. It is a well-established method for assessing trunk strength, with testing protocols in kneeling (Palmer and Uhl, 2011), standing (Andre et al., 2012), and seated positions (Juan-Recio et al., 2017). The seated test protocol specifically isolates lumbar motion and reduces hip force interference. Research has shown that trunk endurance and balance control may not be constraining factors for exceptional performance (Wirth et al., 2022; Keiner et al., 2021). However, some studies have highlighted that the intensity of trunk rotation significantly impacts athletic performance, such as its effect on baseball pitching speed (Taniyama et al., 2021), canoe sprint force (Zinke et al., 2019), and golf swing speed (Gordon et al., 2009). Its role in freestyle swimming remains untested where transverse-plane rotation dominates the roll mechanism (Andersen et al., 2021).

This study examines freestyle swimming (the dominant competitive stroke) using isokinetic dynamometry to evaluate trunk strength-performance relationships. It aims to: (1) quantify trunk strength characteristics across velocities (60°/s, 120°/s) in elite freestyle swimmers; (2) Analyze associations between strength metrics and 100 m sprint performance segments (start, turn, overall). Given the velocity-specific nature of force production in aquatic environments, we hypothesize that (1) peak torque at 120°/s would correlate more strongly with sprint performance than 60°/s, reflecting the high-velocity force demands of the catch phase (Wirth et al., 2022); (2) trunk rotation torque will show a stronger relationship to performance than flexion/extension torque in freestyle, analogous to transverse-dominant sports (Zinke et al., 2019), due to shared roll mechanics.

## Methods

### Participants

Thirty-two elite swimmers (age: 19.49 ± 1.44 years; height: 177.77 ± 6.84 cm; body mass: 71.88 ± 8.50 kg; body mass index: 22.68 ± 1.67 kg/m^2^), including 9 national-level athletes, were recruited for this study after meeting the criteria of being at a Chinese first-class level or higher. The basic information differences existed between sexes as detailed in Table 1. All participants confirmed their lack of significant injuries in the preceding 6 months and provided written informed consent after a comprehensive explanation of the study’s aims and methods. Swimmers usually perform 3-4 swim drills per week and at least 2 structured strength and conditioning sessions per week during the testing cycle. All subjects provided written informed consent and their personal information was handled anonymously. The research protocol was approved by the Ethics Committee of Beijing Sport University.

**TABLE 1:** Baseline data of the subjects.

Basic information	Total (n = 32)	Male (n = 20)	Female (n = 12)
Age (years)	19.49 ± 1.44	19.59 ± 1.71	19.50 ± 1.00
Height (cm)	177.77 ± 6.8	181.11 ± 5.06	172.17 ± 5.84
Body mass (kg)	71.88 ± 8.50	76.06 ± 7.2	65.5 9 ± 7.62
Body mass index (kg/m^2^)	22.68 ± 1.67	23.19 ± 1.77	22.04 ± 1.51
Traing Time (years)	10.27 ± 2.48	9.43 ± 2.31	11.75 ± 2.09
Sprint (50–100 m)	24 (75%)	15 (75%)	9 (75%)
Mid-distance (200–400 m)	5 (15.6%)	3 (15%)	2 (16.7%)
Long-distance (800–1500 m)	3 (9.4%)	2 (10%)	1 (8.3%)

### Experimental design

This study recruited swimmers from Beijing Sport University. Conducted in September 2023, the protocol involved trunk isokinetic muscle strength testing (60–80 min) and 100 m sprint testing (20–30 min) on separate days to mitigate fatigue effects. Participants were acquainted with testing procedures, and their information was documented according to study protocols. To ensure data validity and reliability, subjects completed a warm-up and were advised to avoid strenuous physical activity for 48 h before testing and to fast for 2 h beforehand.

## Procedures

### Isokinetic strength testing

**FIGURE 1:**
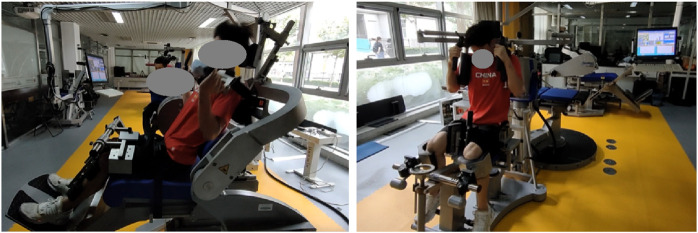
Schematic diagram of trunk flexion/extension and rotation isokinetic testing.

The isokinetic trunk protocol utilized the Isomed 2000 dynamometer (D&R Ferstl GmbH, Hemau, Germany, 2000). Participants were positioned on the dynamometer’s dual-position back extension-flexion attachment with an upright trunk, hips and knees bent at 90°, thighs parallel to the floor, and the dynamometer’s axis aligned with the line between the anterior superior iliac spines, serving as the anatomical reference. Adjustable pads supported the head, sacrum, and upper trunk, with additional pads on the tibia’s anterior surface, secured by Velcro straps on the upper trunk, thighs, and pelvis as shown in Figure 1. Trunk movement was limited to 60°, with 30° of flexion and 60° of extension from the reference position (0°), and rotation testing was confined to 60° range of motion (30° rotation to the left and 30° to the right from the reference position 0°). Hip movement was minimized following standardized stabilization protocols (García-Vaquero et al., 2020). Testing began with flexion/extension trials from neutral, followed by rotation trials starting from the left side. Participants crossed their arms over their chest, with 1–3 min of rest between trials.

Participants were instructed to cross their arms over their chest and exert maximum effort from the start of the first set until the test concluded. Verbal encouragement was provided to ensure maximal effort was sustained throughout testing. Before assessment, participants completed a standardized 15-min warm-up protocol comprising dynamic stretching, core activation exercises, pillar preparation drills, and medicine ball throws. To acclimate to the protocol, participants completed three maximal isokinetic practice contractions followed by ten consecutive maximal efforts. This warm-up facilitated familiarity with the equipment and test procedure. The total testing duration ranged from 60 to 80 min. To accurately assess reliability, each participant underwent five testing sessions of the isokinetic trunk flexion-extension protocol. All trials were conducted at the same time of day and overseen by the same researcher. During the initial testing session, each participant’s position on the dynamometer was logged and consistently controlled across all sets and sessions by adjusting pads and straps to ensure protocol reliability. Peak torque (PT), the maximal moment generated during joint movement, was the primary outcome measure. Testing procedures followed manufacturer guidelines rigorously, administered by certified technicians. Participants received verbal encouragement to exert maximal effort throughout.

#### Sports performance testing

The swimming performance tests were conducted in a 50-m indoor pool maintained at 28°C. Safety protocols included the presence of a standby rescue team. The pool lanes were marked with 50-m spiral float line ropes. Two high-speed waterproof cameras (GoPro HERO7, GoPro Inc., San Mateo, CA, United States) recording at 2.7K and 100 Hz were utilized: one positioned 0.15 m underwater at the lane center for stroke parameter analysis (Franken et al., 2013), and another mobile camera operated by a researcher to capture the entire trial. Stroke time (ST) was measured by a coach using a chronometer, a standard method for identifying stroke parameters (Franken et al., 2013; Khiyami et al., 2022). Prior to the trial, swimmers performed 15 min of warm-up exercises. The performance metrics measured were the total 100 m time, as well as split times at 15 m (start), the turn (5 m pre-turn to 15 m post-turn).

### Statistical analyses

The data are presented as means with standard deviations (SD). Statistical analyses were conducted using IBM SPSS Statistics (version 27; SPSS, Inc., Armonk, NY, United States). Normality of the data was confirmed via the Shapiro-Wilk test, with significance set at *p* < 0.05. Absolute and relative reliability were calculated using the coefficient of variation (CV) and intraclass correlation coefficient (ICC) with absolute agreement (95% confidence intervals), respectively. CV values <10% were considered acceptable (Cormack et al., 2008) and ICC values were interpreted according to the guidelines proposed by Koo and Li (2016), where >0.9 = excellent, 0.75 to 0.9 = good, 0.5 to 0.74 = moderate, and <0.5 = poor. A paired-sample t-test was conducted to examine the trunk muscle strength under different angular velocities and the bilateral muscle strength. Pearson correlations examined relationships between trunk strength measures (8 variables) and swimming performance (3 segments). Given 24 comparisons per subgroup, Benjamini–Hochberg FDR correction were implemented to limit false discoveries to ≤5% of significant results. The procedure identified the largest p-value satisfying pᵢ ≤ (i/24) × 0.05, with all smaller p-values considered significant at q < 0.05.

## Result

The data presented in Table 2 indicate significant sex-based differences in peak torque (PT) for trunk extension and rotation. Trunk extension PT was significantly greater than flexion PT at both high and low angular velocities (p < 0.01). Although left rotation PT was marginally higher than right rotation PT, this difference was not statistically significant. High-speed conditions elicited greater PT in trunk extension and rotation compared to low-speed conditions (p < 0.05).

**TABLE 2:** Mean testing data ± standard deviations for each group.

Test	Total (n = 32)	Male (n = 20)	Female (n = 12)
F 60°/s PT (Nm)	135.42 ± 42.73	155.67 ± 35.23*	100.45 ± 30.59
E 60°/s PT (Nm)	202.60 ± 66.14	241.65 ± 47.34	136.19 ± 31.47
F120°/s PT (Nm)	111.84 ± 26.10	120.84 ± 23.56	96.30 ± 23.58
E 120°/s PT (Nm)	213.10 ± 64.93*	247.42 ± 48.89*	153.82 ± 42.68
LR 60°/s PT (Nm)	122.21 ± 31.09	136.74 ± 26.63	97.12 ± 20.80
RR 60°/s PT (Nm)	118.17 ± 32.51	132.68 ± 29.77	93.09 ± 19.41
LR 120°/s PT (Nm)	130.62 ± 38.13*	152.03 ± 28.13*	93.64 ± 20.37
RR 120°/s PT (Nm)	129.56 ± 40.37*	152.21 ± 30.26*	90.42 ± 20.48
15 m Time (s)	6.80 ± 0.68	6.45 ± 0.39	7.41 ± 0.66
Turn Time (s)	12.13 ± 0.94	11.60 ± 0.60	13.05 ± 0.67
100 m Time (s)	60.07 ± 4.41	57.65 ± 2.09	64.12 ± 4.43

PT, peak torque; F, flexor; E, extensor; LF, left rotation; RR, right rotation.

*: p < 0.05, highlighting differences in angular velocities.

Table 3 displays the peak torque angle distribution of the trunk, showing that peak torque consistently occurs at mid-range angles across all movements, with minimal variability at 60°/s. Notably, there is a significant angle reduction in extensors and rotators at 120°/s, while the flexor angle remains stable but with high standard deviations.

**TABLE 3:** Peak torque angle distribution of trunk (n = 32).

Velocity	Flexor (°)	Extensor (°)	Left rotation (°)	Right rotation (°)
60°/s	17.36 ± 3.84	12.86 ± 5.16	17.66 ± 1.72	17.17 ± 2.65
120°/s	17.04 ± 11.29	5.64 ± 4.63	6.66 ± 4.34	6.24 ± 4.70

Table 4 presents the mean test scores, standard deviations, and reliability metrics, including the coefficient of variation (CV) and intraclass correlation coefficient (ICC). All tests demonstrated acceptable absolute reliability, with CV values under 8%. Relative reliability was excellent, as indicated by ICC values ranging from 0.968 to 0.998.

**TABLE 4:** Accompanying reliability data for each test.

Test	Total (n = 34)	CV (%) (95%CI)	ICC (95%CI)	SEM
F 60°/s PT (Nm)	135.42 ± 42.73	3.86 (3.08–4.77)	0.994 (0.981–0.998)	4.17
E 60°/s PT (Nm)	202.60 ± 66.14	4.65 (3.64–5.97)	0.991 (0.962–0.997)	7.53
F120°/s PT (Nm)	111.84 ± 26.10	5.57 (4.24–6.92)	0.975 (0.954–0.987)	6.90
E 120°/s PT (Nm)	213.10 ± 64.93	7.69 (6.01–9.66)	0.968 (0.941–0.983)	20.54
LR 60°/s PT (Nm)	122.21 ± 31.09	4.33 (3.33–5.41)	0.998 (0.949–0.995)	4.23
RR 60°/s PT (Nm)	118.17 ± 32.51	3.93 (3.04–4.90)	0.989 (0.970–0.995)	4.93
LR 120°/s PT (Nm)	130.62 ± 38.13	5.04 (3.83–6.42)	0.985 (0.973–0.992)	8.05
RR 120°/s PT (Nm)	129.56 ± 40.37	5.37 (4.28–6.70)	0.988 (0.977–0.994)	7.42

PT, peak torque; F, flexor; E, extensor; LF, left rotation; RR, right rotation; CV, coefficient of variation; ICC, intraclass correlation coefficien; CI, confidence interval; SEM, standard error of measurement.

Table 5 presents correlation coefficients between trunk strength and swimming performance segments. Combined sample correlations are provided for reference only and may be confounded by sex differences. After Benjamini–Hochberg FDR correction (q < 0.05), no significant correlations were identified. In male swimmers, trunk strength generally exhibited non-significant or positive correlations with performance metrics showed mixed correlation directions (e.g., 60°/sF-15 m: r = −0.055; 120°/sE-15 m: r = 0.21). The strongest association was 60°/sRR-100 m (r = 0.465, *p* = 0.045), but it did not survive FDR correction. In female swimmers, trunk strength metrics did not show any statistically significant correlations with performance segments. However, early-phase performance (15 m) exhibited moderate negative trends with flexion torque: at 60°/s flexion (r = −0.479) and 120°/s left rotation (r = −0.177), in contrast to the positive correlations observed in males.

**TABLE 5:** Correlation coefficients between trunk strength and performance.

°/s	PT	15 m			Turn-(45–65 m)			100 m		
		Total (n = 32)	Male (n = 20)	Female (n = 12)	Total (n = 32)	Male (n = 20)	Female (n = 12)	Total (n = 32)	Male (n = 20)	Female (n = 12)
60	F	−0.567	−0.055	−0.479	−0.494	0.002	−0.086	−0.461	0.119	−0.185
	E	−0.598	−0.145	−0.1	−0.605	−0.16	0.233	−0.479	0.148	0.247
120	F	−0.376	−0.081	−0.06	−0.375	0.093	−0.262	−0.407	−0.152	−0.055
	E	−0.457	0.21	0.06	−0.567	0.014	−0.008	−0.474	0.076	0.223
60	LR	−0.445	−0.062	−0.04	−0.421	0.088	0.073	−0.325	0.346	0.105
	RR	−0.365	0.174	−0.041	−0.407	0.142	0.069	−0.273	0.465	0.165
120	LR	−0.555	0.107	−0.177	−0.603	0.067	−0.215	−0.511	0.232	−0.045
	RR	−0.547	0.111	−0.161	−0.644	−0.069	−0.273	−0.498	0.34	−0.124

PT, peak torque; F, flexor; E, extensor; LF, left rotation; RR, right rotation.

All correlations non-significant after FDR, correction (q < 0.05).

## Discussion

This study utilized isokinetic dynamometry to assess trunk strength at varying angular velocities and explored its relationship with 100 m freestyle swimming performance. Results indicated that sprint freestyle swimmers exhibited higher peak torque in trunk rotation and flexion during high-speed movements compared to low-speed ones. While swimmers generated greater rotational torque at high speeds (*p* < 0.05), this mechanical advantage did not enhance performance. The lack of FDR-significant associations suggests trunk strength, as measured by isokinetic dynamometry, may not directly determine swimming performance in elite athletes. Training approaches should prioritize inter-segmental coordination, with a focus on optimizing torque transfer from the trunk to the extremities. These findings offer valuable insights for refining training strategies in sports.

The data aggregated across protocols (Table 2) show trunk extension peak torque (PT) significantly exceeded flexion PT at all velocities (*p* < 0.01), with the disparity increasing at higher speeds (e.g., extension-flexion difference: 67.2 Nm at 60°/s vs 101.3 Nm at 120°/s). Consistent with the rapid torque-generation demands in swimming, rotational PT at 120°/s surpassed 60°/s values (*p* < 0.05, Table 2), contrasting the typical velocity-force relationships observed in non-aquatic sports. The angle distribution patterns (Table 3) further revealed that the extensors/rotators exhibited velocity-dependent shifts toward earlier ranges of motion (ROM), with peaks at 5.6°–6.6° at 120°/s compared to 12.9°–17.4° at 60°/s. In contrast, the flexor angles remained stable but with increased variability at 120°/s (SD = 11.29° vs 3.84° at 60°/s), suggesting compromised measurement consistency under high-velocity conditions. These torque-angle profiles align with the biomechanics of freestyle swimming, where rapid torso rotation coordinates with limb propulsion (Ruiz-Navarro et al., 2025), but caution is warranted when interpreting high-velocity data due to the reduced isokinetic phase duration.

The study investigated the relationship between trunk strength and sprint performance. Contrary to hypotheses 1-2, which peak torque at 120°/s shows a stronger correlation with sprint performance than at 60°/s and rotational torque is more closely linked to performance than flexion/extension metrics, no statistically significant correlations were found after correcting for multiple comparisons. The observed nominal associations (p < 0.05 uncorrected) were likely the result of Type I errors due to the large number of statistical tests conducted. While trunk rotation strength has been shown to predict performance in terrestrial rotation-dominant sports like baseball (Taniyama et al., 2021), its transfer to aquatic environments appears limited. Similarly, weak trunk strength-performance links have been reported in soccer (Keiner et al., 2015) and swimming (Prieske et al., 2014), underscoring the context-dependent nature of physical adaptations. Methodological and biomechanical factors may explain the limited relationship between dry-land trunk strength and swimming performance. Isokinetic tests conducted on land cannot replicate the triaxial torque demands experienced during aquatic propulsion (Pérez-Olea et al., 2018). Fluid dynamics favor energy transfer via coordinated body roll over maximal torque output (Zamparo et al., 2012; Ruiz-Navarro et al., 2025). Furthermore, Andersen et al. (2021) found that torso muscles are more crucial for postural stability and control during front crawl swimming than for torso rotation. In conclusion, swimming performance appears to depend more on the technical integration of movement patterns than on isolated trunk strength. The context-specific nature of physical adaptations should be considered when evaluating the relevance of strength measures to aquatic sports performance.

**FIGURE 2:**
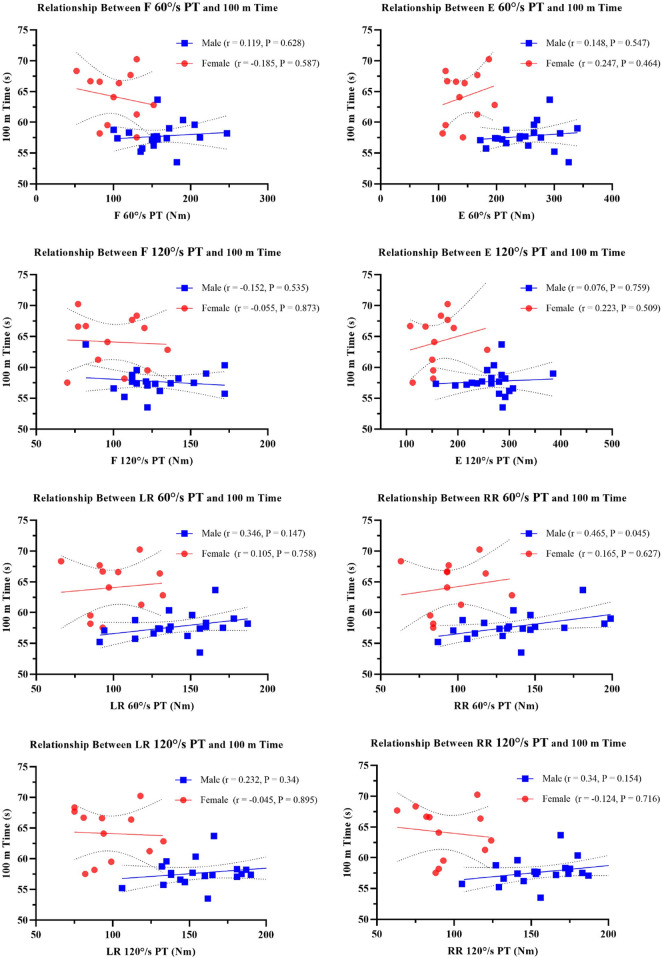
Scatterplots of Trunk Strength versus 100 m Performance PT, Peak Torque; F, flexor; E, extensor; LF, left rotation; RR, right rotation. All correlations non-significant after FDR correction (q < 0.05).

Despite the absence of significant within-sex associations after FDR correction, visual inspection of sex-stratified scatterplots (Figure 2) revealed nominal trends that merit cautious interpretation. Stratified data suggested potential sex-specific patterns: males exhibited a tendency toward positive associations between rotation torque and performance, while females showed a slight trend linking flexion to acceleration and passive relationships between rotation torque and performance. Although these observations are not statistically significant, they suggest the hypothesis of differing kinetic strategies: males might use rotation to compensate for limited sagittal-plane force transmission, while females may focus on flexion-driven streamline control (Andersen et al., 2020). It is critical to emphasize that these patterns did not survive multiple comparisons correction and should be considered strictly exploratory. They serve primarily to highlight the complexity of trunk biomechanics and the necessity for sex-specific investigations in future research with larger samples. Methodologically, pooled correlations were confounded by biological dimorphism, such as males’ generally higher strength and performance. The lack of within-sex associations after stratification confirms that initial combined-sample trends reflected sex differences rather than causal relationships.

The present study challenges the utility of isolated trunk strength as a predictor of elite swimming performance. The non-significant outcomes observed underscore a fundamental disconnect between conventional strength metrics and the biomechanical demands of aquatic locomotion, where hydrodynamic efficiency likely supersedes raw torque output (Zamparo et al., 2012). Within a multifactorial training framework (Wirth et al., 2022), trunk strength should be integrated with technical skills (e.g., stroke efficiency) and physiological capacities, rather than being considered an isolated performance predictor. Importantly, individualized dryland training programs (e.g., velocity-specific loads) can further modulate strength adaptation (Izquierdo et al., 2002; Amaro et al., 2018), potentially explaining the null correlations observed. Several limitations of the current study warrant cautious interpretation. First, peak torque measurements at 120°/s occurred at low joint angles (5.6°–6.5°), which may have captured acceleration artifacts rather than true trunk strength. Second, the male-skewed sampling (M:22, F:12) obscured potential sex-specific differences in the relationships examined. Third, the study did not quantify body roll kinematics or the periodization of dryland training, both of which may have influenced the observed outcomes. Future research should employ instrumented tethered swimming systems to directly quantify trunk force production during aquatic locomotion, synchronizing these measurements with 3D motion capture of roll mechanics. Additionally, sex-stratified analyses in balanced cohorts are needed to identify potential dimorphic strategies. Integrating longitudinal training metrics (e.g., velocity-specific dry-land loads) with comprehensive biomechanical profiling may further elucidate the complex relationships between trunk strength, technical skills, and swimming performance.

The present study utilized isokinetic dynamometry at angular velocities of 60°/s and 120°/s to investigate the relationship between trunk strength and 100 m performance. The findings did not reveal any statistically significant correlations, challenging the notion that isolated trunk strength is a reliable predictor of swimming performance. This suggests that hydrodynamic efficiency in swimming may depend more on inter-segmental coordination and technical efficiency than on maximal torque output alone. Consequently, our study does not support the prioritization of isolated trunk strength training as a performance-enhancing strategy for swimmers. The observed trends between sexes, while intriguing, are exploratory in nature and underscore the need for sex-specific investigations with larger sample sizes in future research.

## Data Availability

The original contributions presented in the study are included in the article/supplementary material, further inquiries can be directed to the corresponding authors.
